# Supplementary material for: Thermal Analysis of Metal-Organic Precursors for Functional Cu:ΝiOx Hole Transporting Layer in Inverted Perovskite Solar Cells: Role of Solution Combustion Chemistry in Cu:ΝiOx Thin Films Processing
Source: Nanomaterials (Basel). 2021 Nov 15;11(11):3074. doi: 10.3390/nano11113074 (PMC8618520; doi:10.3390/nano11113074)
Supplement: Supplementary file 1 [file nanomaterials-11-03074-s001.zip › nanomaterials-1434240-supplementary.pdf]

nanomaterials

MDPI

Supplementary materials

Thermal Analysis of Metal-Organic Precursors for Functional Cu:NiOx Hole Transporting Layer in Inverted Perovskite Solar Cells: Role of Solution Combustion Chemistry in Cu:NiOx Thin Films Processing

Apostolos Ioakeimidis [1], Ioannis T. Papadas [1,2], Eirini D. Koutsouroubi [3], Gerasimos S. Armatas [3] and Stelios A. Choulis [1,*]

[1] Molecular Electronics and Photonics Research Unit, Department of Mechanical Engineering and Materials Science and Engineering, Cyprus University of Technology,3063 Limassol, Cyprus; a.ioakeimidis@cut.ac.cy (A.I.); ioannis.papadas@cut.ac.cy (I.T.P.)
[2] Department of Public and Community Health, School of Public Health, University of West Attica, Athens, Greece
[3] Department of Materials Science and Technology, University of Crete, 70013 Heraklion, Greece; eirinink@materials.uoc.gr (E.D.K.); garmatas@materials.uoc.gr (G.S.A.)
* Correspondence: stelios.choulis@cut.ac.cy

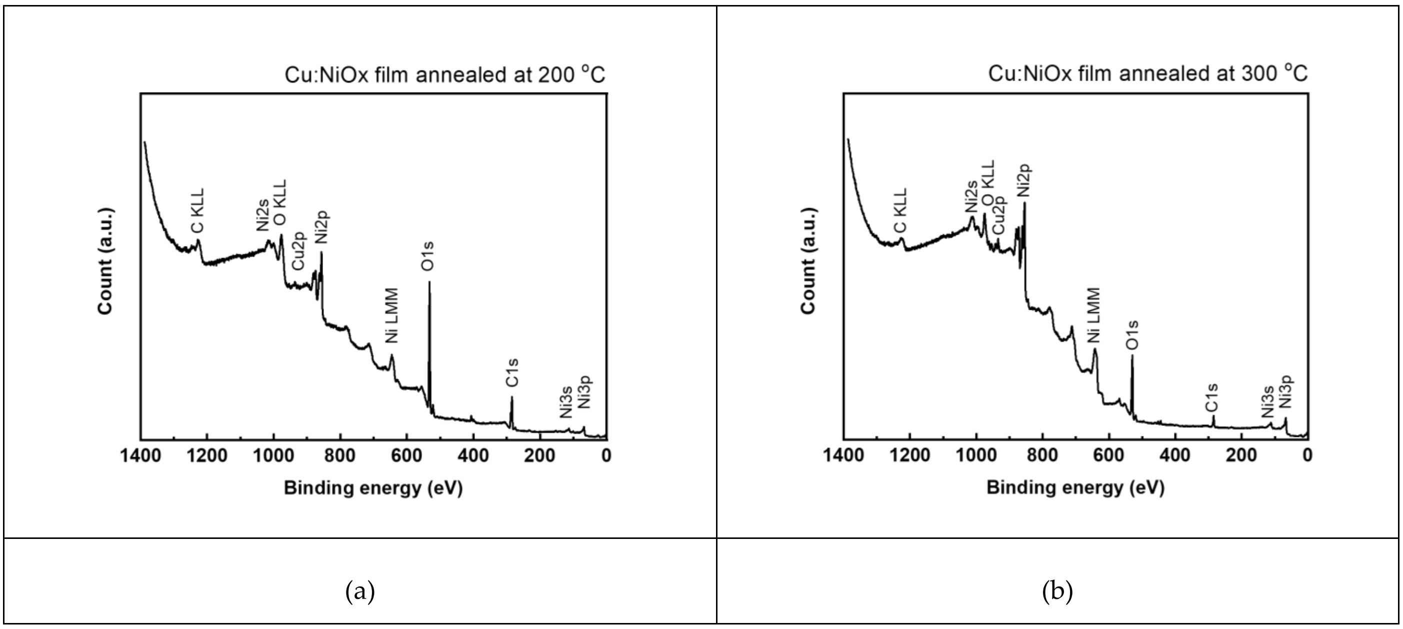

(a)	(b)

Figure S1. XPS survey spectra of the Cu:NiOx films fabricated form precursor containing 0.1 Acac and annealed at (**a**) 200 °C and (**b**) 300 °C.

Table S1. XPS calculated atomic ratios for the Cu:NiOx films fabricated form precursor containing 0.1 Acac and annealed at 200 and 300 °C.

Sample	C[a] (%)	N[b] (%)	Ni (%)	Cu (%)	O (%)
200 °C annealed Cu:NiOx film	18.40	8.05	17.59	2.10	53.86
300 °C annealed Cu:NiOx film	3.26	1.45	42.11	5.22	47.95

[a]C-oxygen/nitrogen bonded species corresponding to a 6.51 and 0.64 wt.% content for 200 and 300 °C annealed Cu:NiOx films, respectively. [b]Nitrogen-containing species corresponding to a 3.32 and 0.33 wt.% content for 200 and 300 °C annealed Cu:NiOx films, respectively.

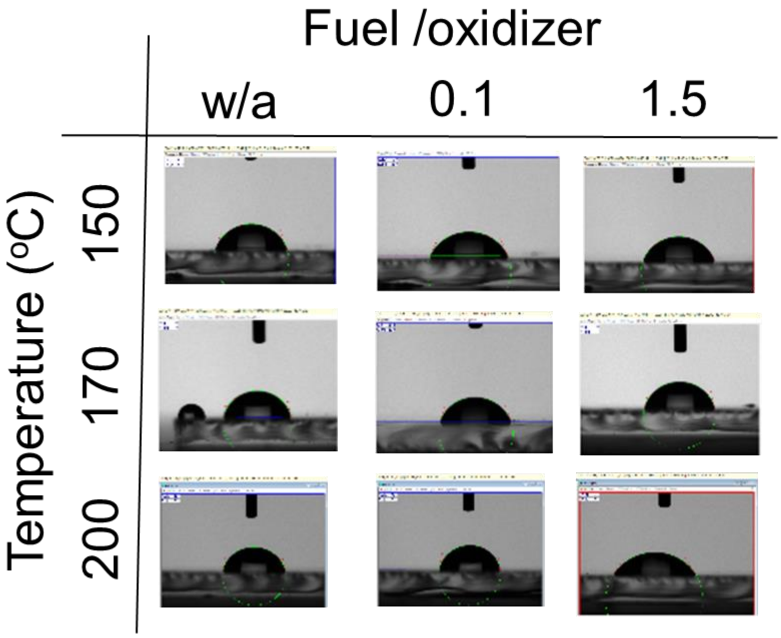

Figure S2. contact angle picture of water on films prepared using precursor containing w/o, 0.1 and 1.5 molar ratio of fuel (Acac) to oxidizer (Cu, Ni nitrates) annealed at 150, 170 and 200 °C, respectively.

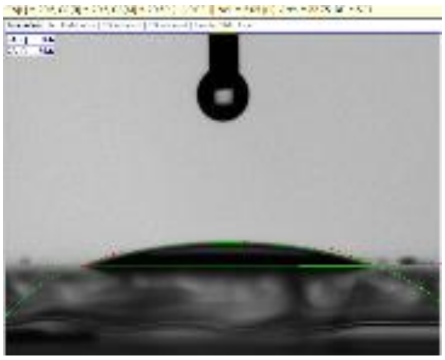

Figure S3. contact angle picture of water on films prepared using precursor 0.1 molar ratio of fuel (Acac) to oxidizer (Cu, Ni nitrates) annealed at 300 °C showing an angle of 20°.
